# Initial Stand Volume and Residual Live Trees Drive Deadwood Carbon Stocks in Fire and Harvest Disturbed Boreal Forests at North‐Central Alberta

**DOI:** 10.1002/ece3.70710

**Published:** 2025-01-16

**Authors:** Richard Osei, Charles A. Nock

**Affiliations:** ^1^ Department of Renewable Resources University of Alberta Edmonton Canada

**Keywords:** deadwood, forest disturbance, island remnants, retention forestry, tree mortality

## Abstract

Retention forestry involves leaving single or groups of unharvested trees within harvest areas. Patch retention, which resembles structures such as unburned patches remaining after wildfire, is one practice implemented within the framework of Ecosystem‐based Forest Management (EBM), which seeks to use natural forests as a model and minimize differences in natural and managed forests. Despite the widespread adoption of patch retention practices, few comparisons of the attributes of postfire and postharvest islands, or their drivers, have been made. Given the importance of deadwood in forests to a variety of ecosystem functions, we sought to compare the local bioenvironmental drivers of deadwood (snags, CWD) C stocks in islands remnants in postfire and postharvest forests a decade after disturbance. We also determined whether their relative effects are consistent across deadwood types (snags, CWD) and disturbance regimes using generalized additive mixed models with study site as random factor in all cases. A candidate model with initial stand volume (ISV), basal area of live trees, and size heterogeneity of live trees best predicted snag and CWD C stocks in both disturbance types, but their relative importance was inconsistent. The ISV had significantly (*p* < 0.05) positive effects on C stocks in snags and CWD across disturbance types, but its relative effects was higher in retention islands than fire islands. In all cases, stand density of remnant live trees was negatively related to deadwood C stocks. Conversely, the size heterogeneity of remnant live trees significantly boosted deadwood C stocks in fire islands but not in harvest islands. The results imply consideration for the stocking level of candidate forest areas for retention patches as this drives the evolution of deadwood accumulation in the postharvest islands.

## Graphical abstract

Drivers of deadwood carbon stocks are elusive, particularly between fire and harvest patches in boreal forests in Alberta. Initial stand volume, basal area of live trees, and size heterogeneity impact deadwood carbon stocks in both disturbance regimes, but their relative effect was inconsistent.

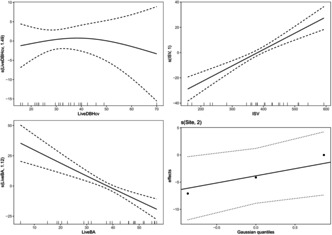

## 1 Introduction

In recent decades, Ecosystem‐based Forest Management (EBM) has emerged as a dominant paradigm at the national and international scale. Central to the concept of EBM is the goal of maintaining or enhancing ecological, economic and social values. EBM is a means to align forest management with the global agenda to reconcile wood extraction with biodiversity conservation and the maintenance of ecological integrity in forests (Gustafsson et al. 2012; Moussaoui et al. 2016a). The retention approach—hereafter structural retention—was introduced 30 years ago (Franklin 1989) and has since been incorporated into forest management practices across the globe (Baker et al. 2013; Fedrowitz et al. 2014; Gustafsson et al. 2012; Lindenmayer et al. 2012; Mori and Kitagawa 2014). At large scales, differences in the frequency, severity, and spatial pattern of disturbances are the focus, whereas at the stand scale, retention of biological legacies (e.g., patches of living trees) represents a key approach (Lindenmayer and Franklin 2002).

Structural retention is based on coarse‐scale emulation of the patterns created by wildfire, which is a dominant form of natural disturbance. After wildfire, biological legacies such as island remnants of un‐burnt forest are generally present within the perimeter of larger burned areas. By leaving structural retention in harvested areas, the aim was to provide continuity and enrichment of structure and in turn maintain key ecological structures and functions; refugia for forest biota, maintenance of coarse woody material (CWM) and live residual trees, wildlife thermal and hiding cover, and corridors for wildlife movement within harvest areas (Alberta Government 2016; Lindenmayer and Franklin 2002). Patches or island remnants are often recognized as key features within forest landscapes disturbed by fire (Andison 2003; Baker, Jordan, and Baker 2016; Gandhi et al. 2004; MacLean et al. 2003). Thus, comparing structure and function of island remnants left within natural disturbances and within harvested areas is key to informing future approaches to the use of structure retention to meet our goals of maintaining resilient forest ecosystems. Deadwood represents a fundamental legacy material with important ecological functions (e.g., biodiversity and C cycling) in the remnant forest landscape for such purposes (Moussaoui et al. 2016b; Nirhamo et al. 2023). Representing about 8% of total forest C stock, deadwood C stocks has become an integral component of forest carbon accounting in recent decades (Martin et al. 2021; Russell et al. 2013). The amount and persistence of deadwood C stock is contingent on local biotic and abiotic factors (Garbarino et al. 2015; Smith, Domke, and Woodall 2022). These include, but not limited to, island size and shape (Hallinger et al. 2016; Jönsson, Weslien, and Gustafsson 2023), topography and climate (Garbarino et al. 2015; Smith, Domke, and Woodall 2022), stand volume of the pre‐disturbance forest (Moussaoui et al. 2016b), and the structure of residual live trees (Garbarino et al. 2015; Hallinger et al. 2016). Although previous studies have evaluated the hierarchy of importance of these factors for deadwood accumulation in specific forests (Garbarino et al. 2015; Smith, Domke, and Woodall 2022), there is no known study that has compared their relative importance in postfire versus postharvest island remnants. Meanwhile, drivers of deadwood C stocks or their relative influence could differ between the two disturbance regimes as a function of potentially contrasting structural attributes of retention and fire islands (Harper et al. 2004; Moussaoui et al. 2016a). The expectation of potential differences is based on the understanding that fire islands were naturally created whereas retention islands are man‐made.

Information on the biotic and abiotic factors dictating the amount and longevity of deadwood C stock is crucial for the preservation of deadwood carbon stocks in managed forests (Tavankar et al. 2022). The objective of the present study was to identify and compare factors driving C stocks in snags (dead standing) and coarse woody biomass (downed deadwood, CWD) between similar‐sized and similar‐aged (~10 years after disturbance) fire‐created versus retention islands.

The study involved deadwood inventory in islands located in boreal mixedwood forests in North‐Central Alberta, estimation of C stocks, and selection of variables for deadwood C stocks based on published literature (Osei et al. 2024). Osei et al. (2024) found similar deadwood carbon stocks between fire and harvest at the study sites but factors driving the carbon stocks remain elusive. Specifically, we tested the hypothesis that local drivers of deadwood C stocks (snags, CWD) differ between postfire islands and retention islands as a result of potentially distinct structure of the remnant forest forests (Harper et al. 2004; Moussaoui et al. 2016a).

## 2 Materials and Methods

### 2.1 The Study Sites and Layout of Plots

**FIGURE 1:**
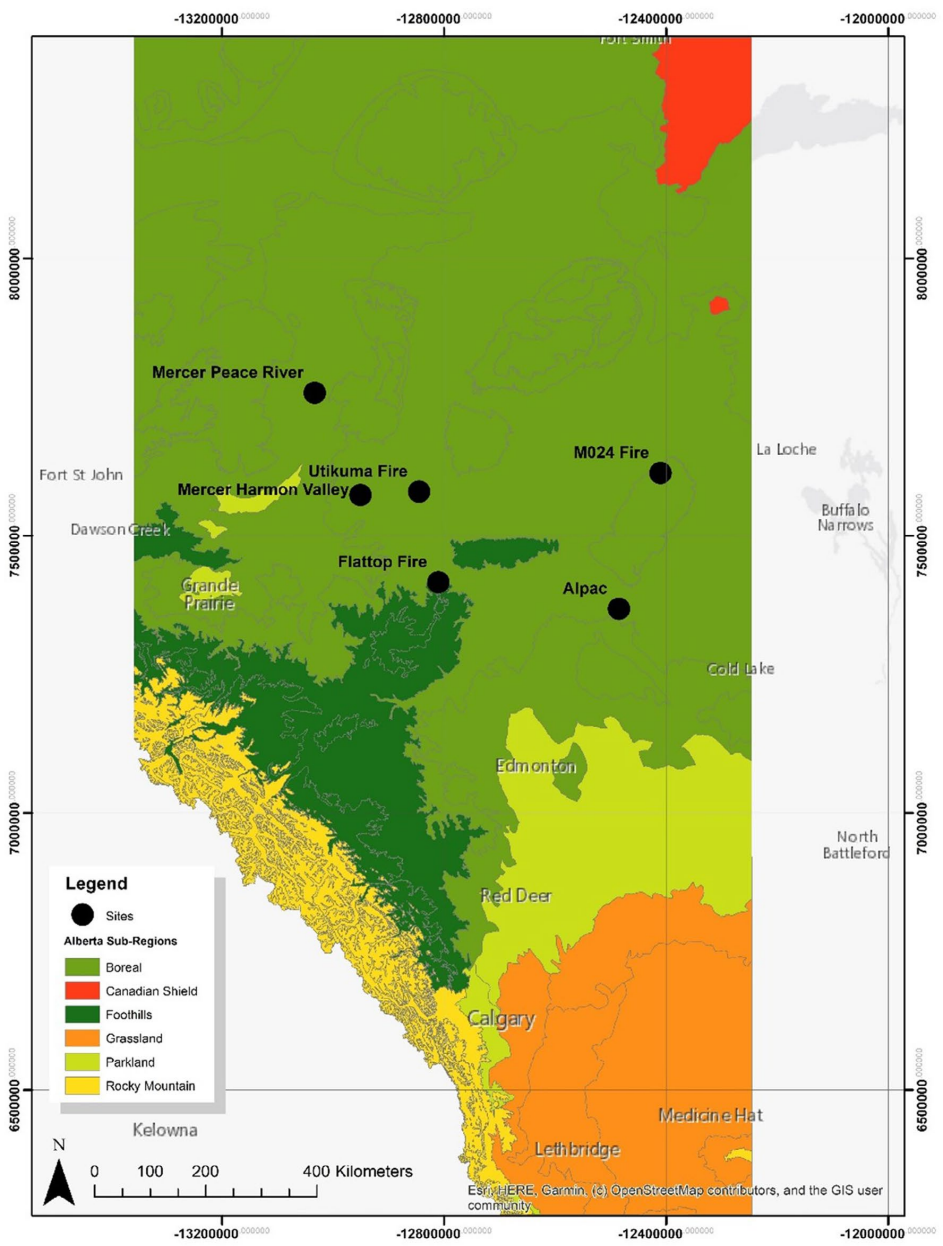
Map of study sites in North‐Central Alberta, Canada.

We conducted this study in the mixedwood boreal forests in North‐Central Alberta. The sites were within the Boreal Plains Ecozone of Canada and were composed of predominantly trembling aspen ( *Populus tremuloides* Michx.; Figure 1). The procedure for site selection and plot layout have been described in Odell et al. (2023). Briefly, we identified a set of unburned forest patches or island remnants in burned forests in the period between 2009 and 2012 via geospatial analyses in ArcGIS and Google Earth. The geospatial analyses were based on the Alberta historical fire polygons, the 2020 Alberta Vegetation Inventory (AVI), and the national dataset of fire burn severity characterized by the delta Normalized Burn Ratio (dNBR). The AVI is a digital vegetation inventory product obtained from aerial photo interpretation that provides information on, among others, tree species composition, stand age, structure, and condition of vegetation in Alberta. We sought to compare wildfire and harvest disturbances that occurred within a similar timeframe to allow comparison of forest attributes (Odell et al. 2023). Upon extensive examination of postharvest island polygons obtained from our industrial partners to unveil potential convergence with available postfire islands created within the same timeframe, we focused on harvested or burned forests between 2009 and 2012 (Odell et al. 2023). The postfire and postharvest island plots were digitized to quantify sizes and selected a subset with similar sizes (0.6–2.7 ha) and species composition. The selected postfire and postharvest island remnants were composed of ≥ 70.0% deciduous species such as *Populus tremuloides, P. balsamifera*, and a small proportion of *Betula* spp. The rest were coniferous species: *Picea glauca* or *Picea mariana* .

We scouted in the field candidate harvest and fire island sites to validate species composition and accessibility before data collection. Afterwards, we chose three harvest sites (Mercer Peace River, Mercer Harmon Valley, Alberta‐Pacific Forest Industries‐Alpac) and three fire sites (Utikuma, Flattop, M024) as shown in Figure 1. Each site consisted of an assemblage of island remnants located close to one another (Table S1). Based on the historical climate data of the Government of Alberta, long‐term average annual precipitation and average annual temperature at the sites were, respectively, within 394.2–493.9 mm/year and 1.1°C–2.0°C (Table S1).

We set up two circular plots with a radius of 11.28 m (~ 400 m^2^ in area) in each island remnant. This size was selected because it is used for the provincial growth and yield initiative (PGYI) in Alberta (Alberta Agriculture and Forestry 2015) and Moussaoui et al. (2016b) used in their study. In all plots, we recorded all standing dead trees (hereafter called snags) with a diameter at breast height (DBH—1.3 m) ≥ 9.1 cm based on the protocol of PGYI (Alberta Agriculture and Forestry 2015). Relying on the Terrestrial Field Data Collection Protocol of the Alberta Biodiversity Monitoring Institute (ABMI 2014), we collected the following information on each snag: DBH, species, and decay class (classes 1–5; high classes show advanced stage of decay). We employed the line‐intercept method (Warren and Olsen 1964) to estimate volume of downed deadwood materials, hereafter, coarse woody debris (CWD). Specifically, we used six transect lines in all circular plots at 0, 60°, 120°, 180°, 240°, and 300° (Warren and Olsen 1964). The criteria for consideration of a piece of wood as CWD were a diameter of ≥ 7.5 cm and length > 1 m. Woody debris that were less than 7.5 cm were excluded following recommendation of Russell et al. (2013) as they are ephemeral, small in size, and there are limited approaches to quantify their carbon stocks. Wood pieces that crossed the line transects were identified to the level of species, genus or the functional group (broadleaf/conifer). After that, we measured CWD diameters and assigned decay classes ranging from 1 (recently killed with all their branches present and bark is intact) to 5 (bark absent with no structural integrity and has no large or fine branches) (ABMI 2014; Maser et al. 1979).

### 2.2 Estimation of Deadwood Carbon Stocks

The Species‐specific allometric equations available in Lambert, Ung, and Raulier (2005) were used to first estimate biomass of individual snags (in Kg, then to Mg). We estimated C in each snag by multiplying snag biomass with the species and decay class specific C concentrations in Strukelj et al. (2013). For each plot, we added carbon values from snags in decay class 1 and 2, and then divided by plot area (0.04 ha) to obtain recent snag C stock (Mg C/ha) based on the study by Angers, Bergeron, and Drapeau (2012) in Quebec, Canada. Likewise, we added snag C in decay classes 3–5 and divided by plot area to obtain old snag C stock (Mg C/ha). Total snag C stocks were the sum of recent and old snag C stocks at the plot level. We calculated the volume of individual CWDs by the simplified Huber's formula, which is based on diameters of CWDs measured at the point of intersection as in Marshall, Davis, and Taylor (2003). Individual volumes of CWDs were multiplied by species and decay class specific wood density available in Strukelj et al. (2013) and Köster et al. (2015) to get CWD biomass (Kg/ha; then converted to Mg/ha). We multiplied individual CWD biomass by species and decay class specific C concentration values in Strukelj et al. (2013) and Köster et al. (2015) to obtain CWD C stock (Mg C /ha). We aggregated CWD C stock per transect, and the plot‐level recent, old, and total CWD C stock (Mg C/ha) was calculated as the average of the corresponding values from the six line intersects installed in each circular plot. For a small proportion of deadwood materials in advanced stages of decay, we could not identify to species or genus level. Thus, we used the wood density and C concentration values of aspen because it represented more than 60% of the deadwood materials we could identify.

### 2.3 Statistical Analyses

Our analyses focused on local drivers of deadwood carbon stock and whether their relative effects on deadwood carbon stocks differ by disturbance type. Based on an extensive review of published research and consideration of our study site context, we selected six variables known to influence deadwood carbon dynamics in post‐disturbance forests (Table S2). As described in Table S2, the variables included island area and shape index to indicate island characteristics, initial stand volume (ISV) before disturbance, organic litter depth to characterize site moisture and tree anchorage, total basal area of live trees and their variability in size (i.e., DBH) to characterize the structure of remnant live trees. The six factors were all statistically similar between the two disturbance types based on Welch's Two Sample *t*‐tests (Table S2). Subsequently, we developed five ecologically plausible candidate generalized additive mixed models (GAMM), which allowed fitting the explanatory variables as smoothing terms to unveil possible nonlinear relationships with the response variables and compared them based on the Akaike information criteria corrected for sample size (AICc; Burnham and Anderson 2002; Table S3). All candidate models included study site as a random factor due to sampling multiple plots at a given site, and to also control the potential effects of confounding site factors. We included ISV in all candidate models (Table S3) because of their recorded effects on deadwood accumulation in the literature (Garbarino et al. 2015; Moussaoui et al. 2016b; Smith, Domke, and Woodall 2022). The first candidate model was based on ISV and island characteristics, and the second model had organic litter depth added to the parameters in the first model:

(1) response~shape index+island area+ISV

(2) response~shape index+island area+ISV+organic litter depth

The third model was based on size variability of live trees (LiveDBHcv), total basal area of live trees (LiveBA), and ISV; the fourth model had organic litter depth added to the third model.

(3) response~LiveDBHcv+LiveBA+ISV

(4) response~LiveDBHcv+LiveBA+ISV+organic litter depth

The final model included all the six local variables considered in this study:

(5) $$\begin{array}{c} \text{response}\sim \text{shape index}+\text{Island area}\\ +\mathrm{ISV}+\text{Live DBHcv}+\text{LiveBA}+\text{organic litter depth} \end{array}$$

In all models, the responses were carbon stocks in total snag, recent snags, total CWD, and recent CWD. We used the mgcv R package (Wood, 2017) to fit GAMMs and verify concurvity (i.e., GAMM version of collinearity) among the explanatory variables. In the GAMM, we set the number of knots for the splines all variables at a low value of 3 to curtail overfitting. We used DHARMa R package (Hartig 2022) to perform model diagnostics to confirm adequacy of the models (Figure S1). To assess the relative importance of explanatory variables in the best model (i.e., Model 3; Table S3), we calculated the change in model deviance with and without each explanatory variable (i.e., full model deviance—model deviance without variable of interest) and summed the values recorded for all variables. We converted each variable's contribution to the aggregated deviances to percentages as proxies for relative importance. Therefore, the variable whose exclusion from the selected model reduced total model deviance the most was considered the most influential driver of the response. We evaluated significance of effects at 95% confidence level in all models.

## 3 Results

**FIGURE 2:**
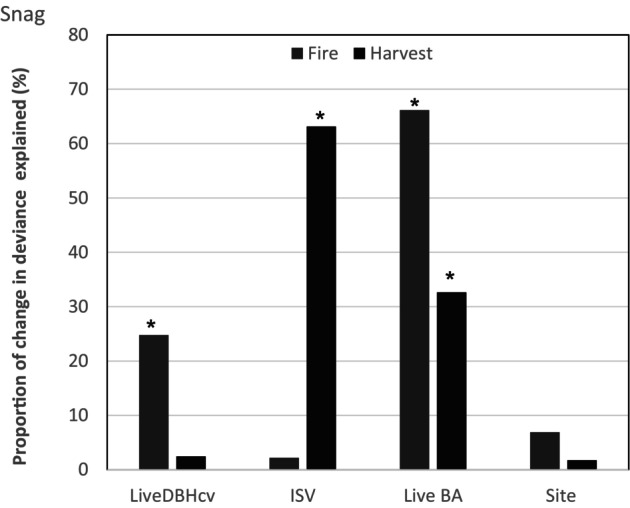
Drivers of total snag C stocks and their relative importance in the two disturbance types. LiveDBHcv is the coefficient of variation of live trees' diameters (%), ISV is the initial stand volume (m^3^/ha), LiveBA is the total basal area of live trees (m^2^/ha) as fixed effects. Site represents the study locations fitted as random effect in the model. Bars with asterisks are significant at 95% confidence level. See Table 1 for full statistical results.

**FIGURE 3:**
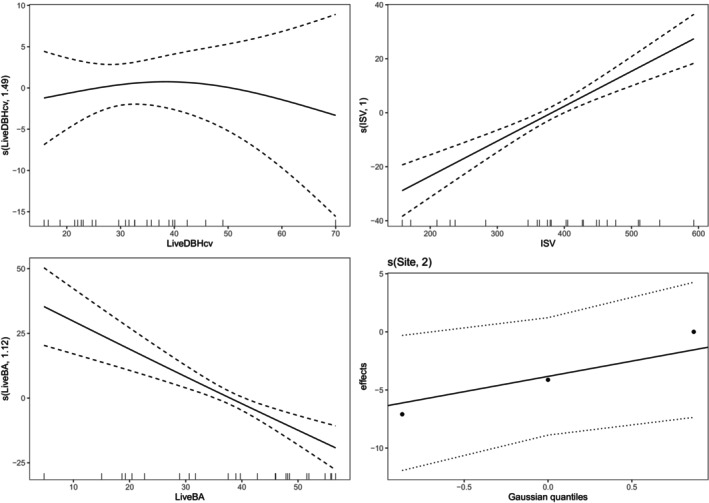
Partial effect plots showing the component effect of GAMM smooth terms on snag C stock in retention islands. LiveDBHcv is the coefficient of variation of live trees’ diameters (%), ISV is the initial stand volume (m^3^/ha), LiveBA is the total basal area of live trees (m^2^/ha) as fixed effects. Site represents the study sites fitted as random effect in the models. See Table 1 for full statistical results.

Comparison of the five candidate models showed that Model 3 was the best for all response variables in both disturbance types (Table S2). Consequently, further analyses focused on the impact of total basal area of live trees, size variability of live trees, and ISV on deadwood carbon stocks. The main drivers of deadwood C stocks were the ISV and the basal area of live trees in both disturbance types (Table 1). The size heterogeneity of live trees had significant effects on C stocks in snags in postfire island remnants but not in postfire island remnants (Table 1; Figure 2). It accounted for 24.7% variability in snag C stocks found in postfire island remnants, and this was 10 times the variability in snag C stock explained in postharvest islands (Figure 2). Furthermore, predictions from the GAMM illustrated an increase in snag C stocks as size variability of live trees increased (Figure S2). On the contrary, ISV was a significant predictor of snag C stocks in postharvest islands (*p* < 0.001) but had no effects on snag C stocks in postfire islands (Table 1). Accordingly, ISV was about 30 times more important for snag C stocks in postharvest island remnants than in postfire island remnants (Figure 2). Additionally, ISV was positively associated with snag C stocks in postharvest island remnants (Figure 3). As opposed to ISV and size structure of live trees that had inconsistent effects on snag C stocks in both disturbance types, the basal area of live trees significantly influenced snag C stocks in both disturbance types (Table 1). However, basal area of live trees was twice as important for snag C stocks in postfire islands as in postharvest islands (Figure 2). In both disturbance types, it was negatively related to predicted snag C stocks (Figure 3; Figure S2).

**TABLE 1:** Results of generalized mixed model with study site as random effect and coefficient of variation of live trees' diameters (LiveDBHcv; %), initial stand volume (ISV, m3/ha), and total basal area of live trees (LiveBA; m^2^/ha) as fixed effects. We report for each driver the estimated degrees of freedom, *F* values, *p* values. Significant *p* values are in bold.

Driver	Snag						CWD					
	Fire			Harvest			Fire			Harvest		
	df	*F*	*p*	df	*F*	*p*	df	*F*	*p*	df	*F*	*p*
LiveDBHcv	1.0	6.5	**0.01**	1.48	0.4	0.64	1.9	13.4	**< 0.001**	1.58	1.48	0.34
ISV	1.0	0.35	0.55	1.0	37.2	**< 0.001**	1.9	21.3	**< 0.001**	1.0	26.6	**< 0.001**
LiveBA	1.0	9.6	**0.01**	1.1	17.3	**< 0.001**	1.0	25.7	**< 0.001**	1.8	10.2	**< 0.001**
Site	2.0	0.77	0.47	2.0	1.3	1.3	2.0	0.01	0.99	2.0	1.3	0.28
Deviance (%)	51.8			81.3			87.9			85.6		

**FIGURE 4:**
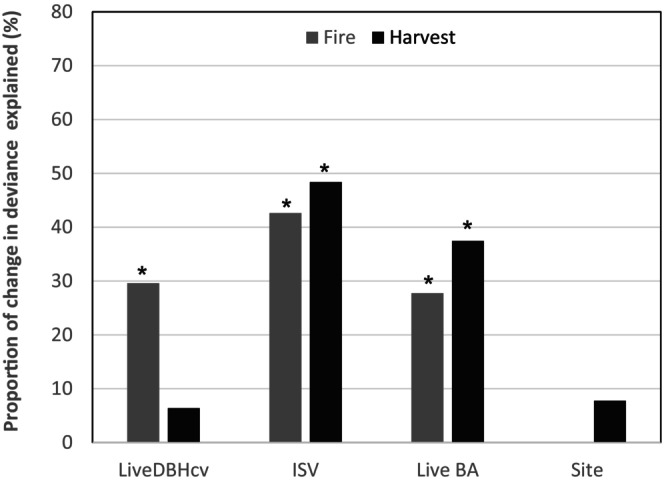
Drivers of CWD carbon stocks and their relative importance in the two disturbance types. LiveDBHcv is the coefficient of variation of live trees' diameters (%), ISV is the initial stand volume (m^3^/ha), and LiveBA is the total basal area of live trees (m^2^/ha) as fixed effects. Site represents study sites fitted as random effect in the models. Bars with asterisks are significant at 95% confidence level. See Table 1 for full statistical results.

**FIGURE 5:**
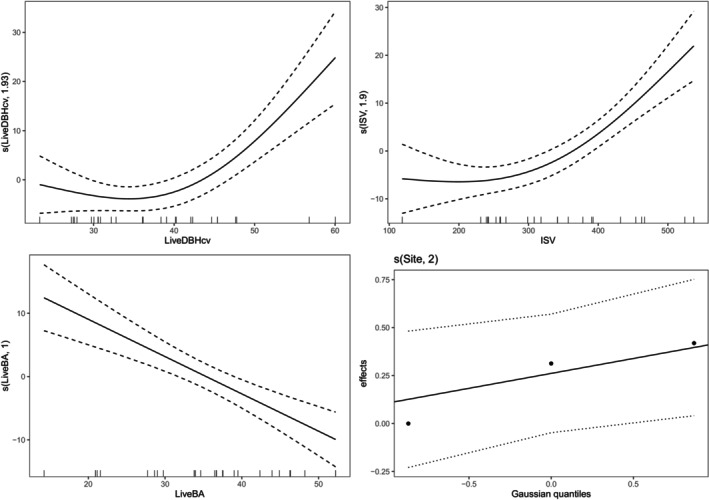
Partial effect plots showing the component effect of GAMM smooth terms on CWD C stock in fire islands. LiveDBHcv is the coefficient of variation of live trees' diameters (%), ISV is the initial stand volume (m^3^/ha), and LiveBA is the total basal area of live trees (m^2^/ha) as fixed effects. Site represents study sites fitted as random effect in the models. See Table 1 for full statistical results.

The basal area of live trees and ISV were important drivers of CWD carbon stocks in both harvest and fire disturbed areas (Table 1; Figure 4). Conversely, tree size variability impacted CWD C stocks only in postfire island remnants (Table 1; Figure 4). The size distribution of live trees was 4.6 times more important for CWD C stock in postfire island remnants than in postharvest remnants (Table 1; Figure 4). Similar to its relationship with snag C stocks, predicted CWD C stocks in postfire island remnants increased with size complexity of live trees (Figure 5).

In contrast with snag C stocks, the importance of ISV and the basal area of live trees for CWD C stocks were apparently similar in both disturbance regimes (Figure 4). Although ISV promoted the accumulation of CWD C stocks in both disturbance types, increase in the basal area of live trees reduced CWD C stocks in both disturbance types (Figure 5; Figure S3). Further investigations revealed that the pattern of impact of the studied variables on total snag and CWD C stocks in both disturbance types was replicated on C stocks in recent deadwood that died post‐disturbance (Table S4).

## 4 Discussion

This study tested the hypothesis that local drivers of deadwood C stocks (snags, CWD) differ between postfire and retention island remnants in boreal mixedwood forests of Alberta, Canada. We found that a candidate model comprising of ISV, total basal of live trees, and size variability of live trees best predicted deadwood C stocks for both fire and harvest disturbed areas. However, the relative importance of these factors for deadwood C stock was dependent on the deadwood type (snag, CWD) and disturbance regime under consideration. We have discussed these findings below.

The model with ISV, total basal area of live trees, and size heterogeneity of live trees being the best of the candidate models for both deadwood types and the two disturbance types suggest that key drivers of deadwood C stocks are somehow similar between postfire and postharvest island remnants, albeit with varying magnitude of effects. With the exception of snag C stocks in postfire islands, ISV had significant effects on deadwood C in all other cases across disturbance types. This supports findings from previous studies that the initial volume of trees per unit forest area is a key determinant of the evolution of deadwood accrual patterns in post‐disturbance landscapes (Garbarino et al. 2015; Moussaoui et al. 2016b; Smith, Domke, and Woodall 2022). In the study by Moussaoui et al. (2016b), ISV was consistently selected in all the top models predicting recent deadwood volume in eastern Canada's boreal forests. The observed positive relationship between ISV and deadwood carbon, when significant, results from the possibility of high deadwood recruitment in forests with high ISV prior to disturbance events (Garbarino et al. 2015; Moussaoui et al. 2016b). The significant positive effects of ISV on CWD C in both disturbance types stems from the fact that disturbance‐induced snag fall represents the main recruitment source to the CWD C pool so islands with high ISV guarantees greater input flux of snags than islands with low ISV (Garbarino et al. 2015). Interestingly, ISV had significant effect on snag C stocks in retention but not in postfire islands, although ISV was statistically similar between the two disturbance types (Welch's *t*‐test; *t* = −1.82, df = 47.08, *p* value = 0.07; Table S2). This points to the possibility that transition of standing live trees into snags after fire events is likely dependent on fire severity as opposed to prefire forest stocking (Smith, Domke, and Woodall 2022).

The basal area per hectare of live trees, an indicator of stand density, was a significant driver of snags and CWD C stocks found in both disturbance types. In all cases, stand density of live trees had negative effects on deadwood C stocks as found in (Moussaoui et al. 2016a). This is similar to that of Hallinger et al. (2016) where increase in stand density reduces tree mortality, and thus reduces snag C stocks. Relatively dense islands remnants could reduce tree mortality, and thus deadwood accrual, by sheltering trees against winds (Hallinger et al. 2016; Larocque et al. 2013) or reduce deadwood decomposition via canopy shading effects (Hallinger et al. 2016). It is noteworthy, however, that self‐thinning could be triggered when a certain level of stand density of live trees is reached thereby increasing tree mortality and deadwood C stocks (Rosenvald and Lõhmus, 2008). In agreement with our expectation, the magnitude of effects of basal area of live trees on snag C stocks was stronger in fire islands than retention islands possibly because fire landscapes are often open and wind‐sheltering effects are more important than in retention islands (Harper et al. 2004; Moussaoui et al. 2016a; Nirhamo et al. 2023).

Along the same lines, our results show that an increase in size inequality of remnant live trees (i.e., LiveDBHcv) was positively related to snag and CWD C stock in postfire Island but had no significant impact on deadwood C stock in postharvest islands. It is particularly intriguing given that size heterogeneity of live trees was similar between fire and retention island remnants (Welch's *t*‐test; *t* = 1.64, df = 42.09, *p‐*value = 0.10). Previous studies have indicated that fires can have a more substantial and lasting impact on the structure of remnant forests while the structure of preharvest forest is mostly maintained in postharvest islands (Harper et al. 2004; Moussaoui et al. 2016a; Nirhamo et al. 2023). It is, therefore, not surprising that the structure of the remnant live trees would play an influential role in ecosystem processes such as tree mortality and deadwood accumulation in structurally altered postfire forest systems compared with structurally intact postharvest islands (Harper et al. 2004). The positive effects in postfire islands may result from the fact that high size variability enhances horizontal and vertical space filling and thus forest productivity in postfire islands (Harper et al. 2004), which results in more live tree biomass per unit area and, consequently, more deadwood inputs (Garbarino et al. 2015; Moussaoui et al. 2016a; Smith, Domke, and Woodall 2022).

## 5 Conclusion

This study investigated whether local drivers of deadwood (snags, CWD) carbon stock differ between retention and fire island remnants in North‐Central Alberta, Canada. A candidate model with ISV, basal area of live trees, and size heterogeneity of live trees best characterized deadwood carbon stock in both disturbance types but their relative importance of was inconsistent. This implies that the actual drivers of deadwood carbon stocks in both disturbances are largely similar although their magnitude of effects is not uniform across disturbance types. The positive effect of ISV on deadwood carbon stock suggests that selection of fairly stocked forests stands as retention islands could guarantee gradual accumulation of deadwood over time. This approach could also safeguard saproxylic biodiversity and other ecological roles of deadwood in the postharvest forest landscape.

## Author Contributions

**Richard Osei:** conceptualization (supporting), data curation (lead), formal analysis (lead), investigation (lead), methodology (supporting), visualization (lead), writing – original draft (lead), writing – review and editing (equal). **Charles A. Nock:** conceptualization (lead), data curation (supporting), formal analysis (supporting), funding acquisition (lead), investigation (equal), methodology (lead), project administration (lead), resources (lead), software (lead), supervision (lead), writing – original draft (supporting), writing – review and editing (supporting).

## Conflicts of Interest

The authors declare no conflicts of interest.

## Data Accessibility Statement

The data for this research have been uploaded as supporting information.

## Supporting information


Data S1.

Data S2.

## Data Availability

The data for this research has been uploaded as supporting information.
