# Supplementary material for: Initial Stand Volume and Residual Live Trees Drive Deadwood Carbon Stocks in Fire and Harvest Disturbed Boreal Forests at North‐Central Alberta
Source: Ecol Evol. 2025 Jan 16;15(1):e70710. doi: 10.1002/ece3.70710 (PMC11738640; doi:10.1002/ece3.70710)
Supplement: Supplementary file 1 — Data S1. [file ECE3-15-e70710-s002.docx]

**SUPPLEMENTARY MATERIAL**

Table S1: Location and characteristics of the harvest (Mercer Peace River, Mercer Harmon Valley, Alpac) and fire (Utikuma, Flattop, MO24) disturbed study sites in this study (Mean Annual Precipitation, MAP; Mean Annual Temperature, MAT), and the number of islands selected. The climate data was obtained from the Government of Alberta Historical Climate Data (1997-2021).

| Site | Latitude | Longitude | MAP (mm/yr) | MAT (°C) | Island remnants |
| --- | --- | --- | --- | --- | --- |
| Mercer Peace River | 56.9886 | -117.0746 | 394.2 | 1.6 | 3 |
| Mercer Harmon Valley | 56.0751 | -116.3352 | 436.2 | 1.9 | 5 |
| Alpac | 55.0318 | -112.1518 | 473.1 | 1.7 | 4 |
| Utikuma Fire | 56.1068 | -115.3864 | 433.2 | 1.7 | 4 |
| Flattop Fire | 55.2781 | -115.0726 | 465.6 | 2.0 | 6 |
| M024 Fire | 56.2752 | -111.4792 | 493.9 | 1.1 | 3 |

**Table S2:** Potential drivers of deadwood carbon stock based on previous studies.

| **Driver** | **Mean (range):**  **Fire** | **Mean (range):**  **Harvest** | **Description** | **References** |
| --- | --- | --- | --- | --- |
| IslandArea | 1.5^a^  (1.0 – 2.2) | 1.4^a^  (0.6- 2.7) | Area of island remnant (ha) | Hallinger et al. (2016);  Jönsson et al. (2023);  Moussaoui et al. (2016b) |
| ShapeIndex | 1.4^a^  (1.1 – 1.6) | 1.2^a^  (1.0 - 1.4) | Island shape complexity;  0.25*perimeter/√area | Hallinger et al. (2016);  Jönsson et al. (2023);  Moussaoui et al. (2016b) |
| ISV | 324.3^a^  (118.5 - 537.1) | 381.3^a^  (158.2 - 592.9) | Initial stand volume (m^3^/ha); live trees + deadwood in class 1 & 2 | Moussaoui et al. (2016b) |
| OrgDepth | 7.0^a^  (2.6 - 13.3) | 7.6^a^  (2.3 - 14.6) | Organic layer depth (cm) to denote tree anchoring substrate and site moisture. | Garbarino et al. (2015);  Moussaoui et al. (2016b) |
| LiveDBHcv | 37.5^a^  (23.3 - 60.0) | 32.5^a^  (15.7 - 69.9) | Coefficient of DBH variation of live trees in a plot (%) | Garbarino et al. (2015); Smith et al. (2022) |
| LiveBA | 35.3^a^  (14.2 - 52.2 | 38.0^a^  (4.7 - 56.6) | Basal area of live trees per ha (m^2^/ha) to represent stand density | Garbarino et al. (2015); Smith et al. (2022) |

Table S3: The AICc values of candidate models compared to select the best for driving snag and CWD C stocks. Recent deadwood are those in decay class 1 and 2; total refers to all snags or CWD irrespective of decay class.

| **Post-fire remnants:** Snags | Recent | Total |
| --- | --- | --- |
| Model 1: response ~ Shape index + Island area + ISV | 197.83 | 238.38 |
| Model 2: response ~ Shape index + Island area + ISV + Organic depth | 201.87 | 235.20 |
| Model 3: response ~ Live DBH cv + Live BA + ISV | **192.42** | **220.69** |
| Model 4: response ~ Live DBH cv + Live BA + ISV + Organic depth | 193.96 | 223.21 |
| Model 5: response ~ Shape index + Island area + ISV + Live DBH cv + Live BA + Organic depth | 205.12 | 226.80 |
|  |  |  |
| **Post-fire remnants:** CWD | Recent | Total |
| Model 1: response ~ Shape index + Island area + ISV | 202.96 | 226.42 |
| Model 2: response ~ Shape index + Island area + ISV + Organic depth | 205.93 | 225.74 |
| Model 3: response ~ Live DBH cv + Live BA + ISV | **184.76** | **192.22** |
| Model 4: response ~ Live DBH cv + Live BA + ISV + Organic depth | 187.52 | 196.64 |
| Model 5: response ~ Shape index + Island area + ISV + Live DBH cv + Live BA + Organic depth | 200.41 | 210.07 |
|  |  |  |
| **Post-harvest remnants:** Snags | Recent | Total |
| Model 1: response ~ Shape index + Island area + ISV | 170.36 | 188.71 |
| Model 2: response ~ Shape index + Island area + ISV + Organic depth | 174.533 | 192.24 |
| Model 3: response ~ Live DBH cv + Live BA + ISV | **156.64** | **169.01** |
| Model 4: response ~ Live DBH cv + Live BA + ISV + Organic depth | 162.3161 | 174.79 |
| Model 5: response ~ Shape index + Island area + ISV + Live DBH cv + Live BA + Organic depth | 176.95 | 180.27 |
|  |  |  |
| **Post-harvest remnants**: CWD | Recent | Total |
| Model 1: response ~ Shape index + Island area + ISV | 172.64 | 184.03 |
| Model 2: response ~ Shape index + Island area + ISV + Organic depth | 172.54 | 184.61 |
| Model 3: response ~ Live DBH cv + Live BA + ISV | **144.05** | **170.55** |
| Model 4: response ~ Live DBH cv + Live BA + ISV + Organic depth | 145.65 | 175.83 |
| Model 5: response ~ Shape index + Island area + ISV + Live DBH cv + Live BA + Organic depth | 162.563 | 197.50 |


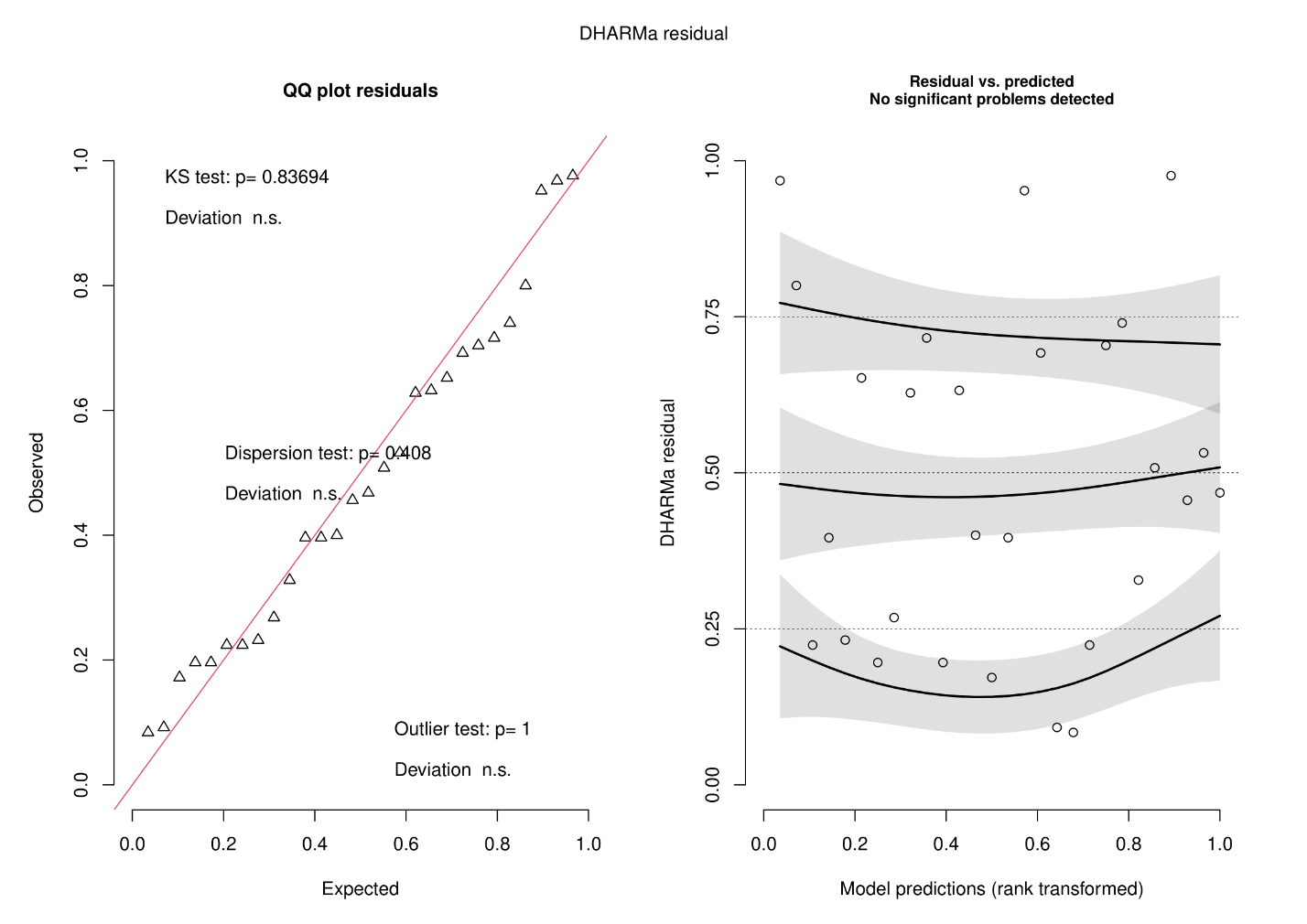


Fig. S1: Example of diagnostics performed for all GAMM to determine their adequacy using the *DHARMa* R package (Hartig, 2022)


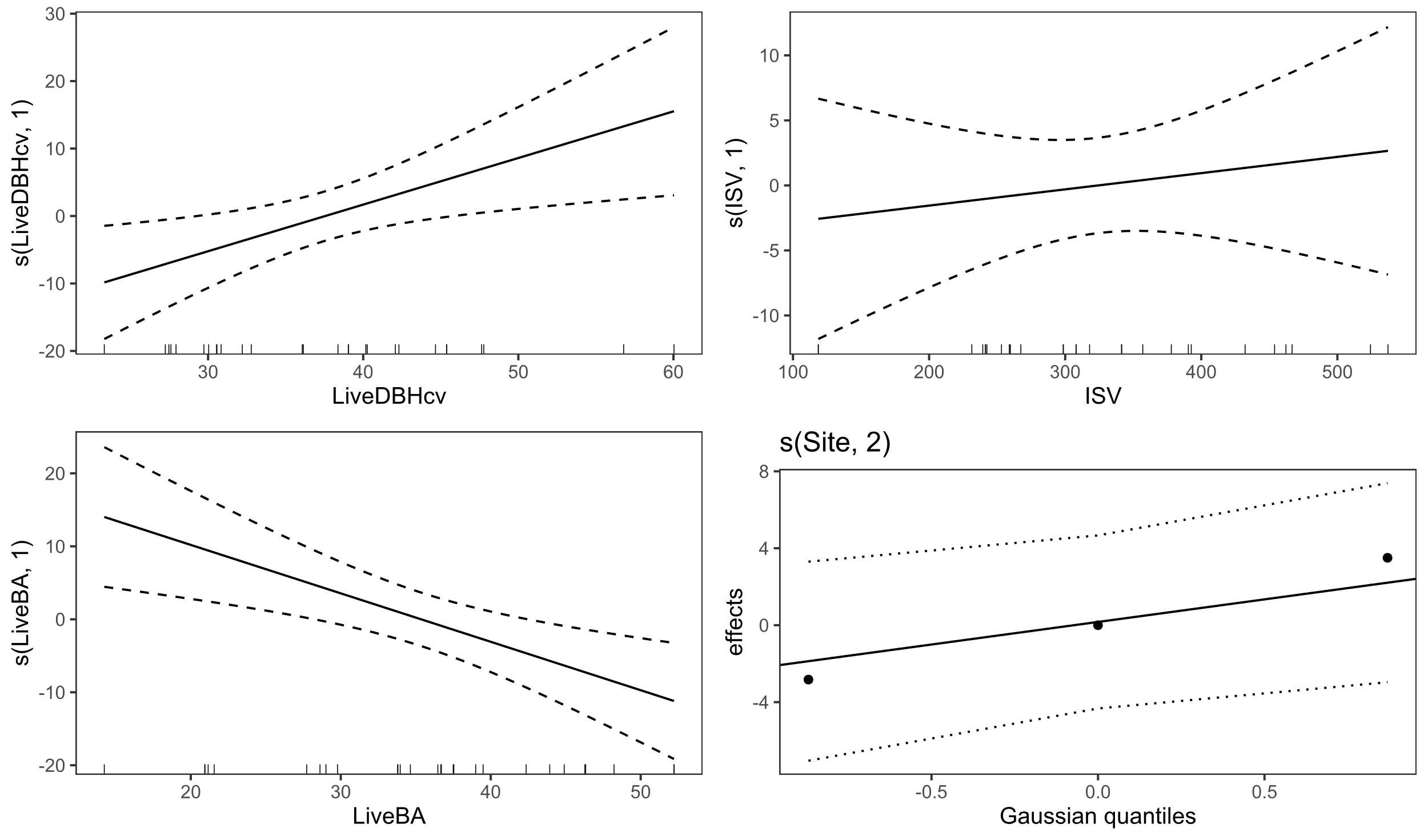


Fig. S2: Partial effect plots showing the component effect of GAMM smooth terms on snag C stock in fire islands. LiveDBHcv is the coefficient of variation of live trees’ diameters (%), ISV is the initial stand volume (m^3^/ha), LiveBA is the total basal area of live trees (m^2^/ha) as fixed effects. Site represents the study sites fitted as random effect in the models. See Table 1 for full statistical results.


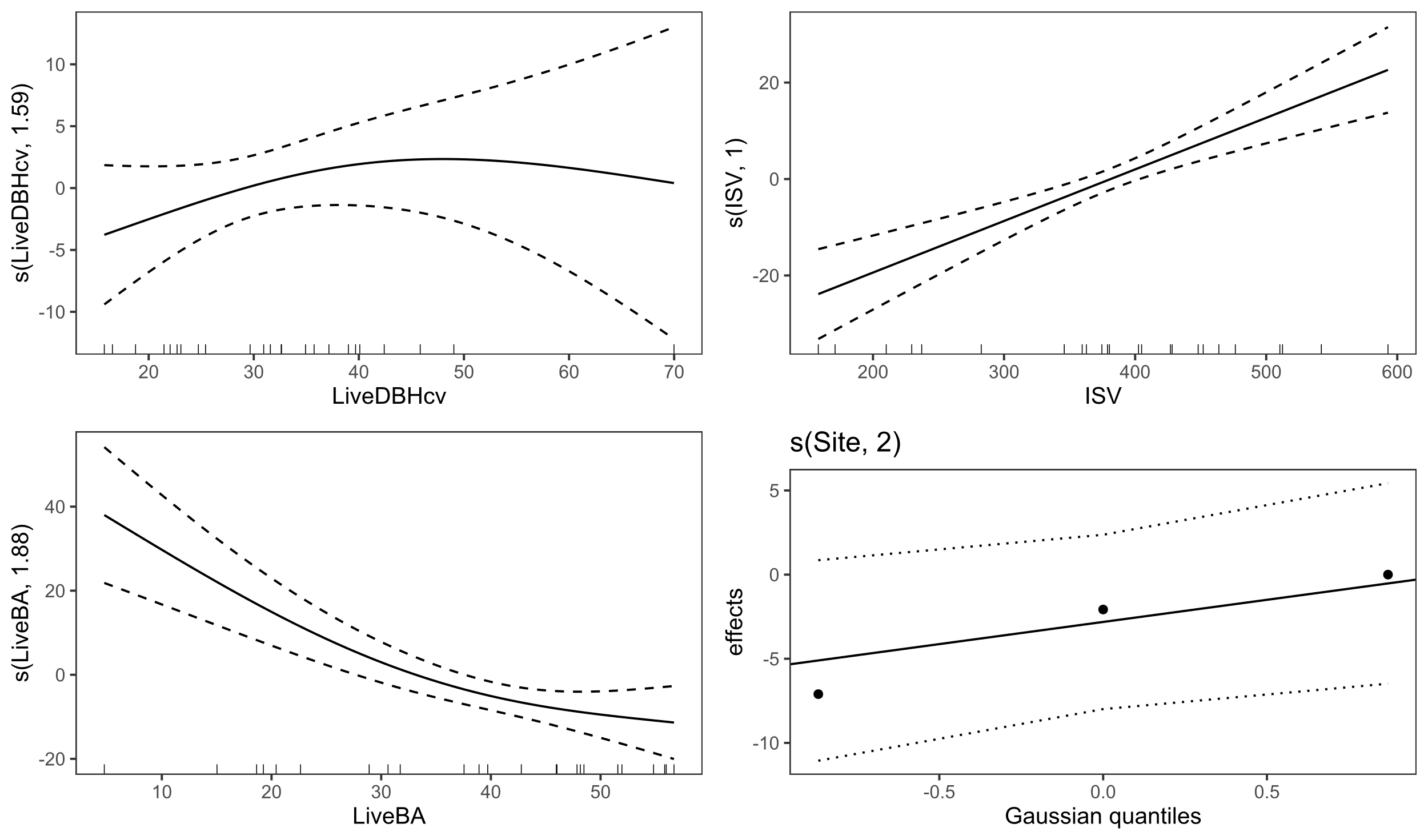


Fig. S3: Partial effect plots showing the component effect of GAMM smooth terms on CWD C stock in retention islands. LiveDBHcv is the coefficient of variation of live trees’ diameters (%), ISV is the initial stand volume (m^3^/ha), LiveBA is the total basal area of live trees (m^2^/ha) as fixed effects. Site represents the study sites fitted as random effect in the models. See Table 1 for full statistical results.

Table S4: Results of generalized mixed model with study site as random effect and coefficient of variation of live trees’ diameters (LiveDBHcv; %), initial stand volume (ISV, m3/ha), total basal area of live trees (LiveBA; m2/ha) as fixed effects for recent snag and CWD C stocks. We report for each driver the estimated degrees of freedom, F-values, p-values. Significant p-values are in bold. Significant p-values (at 95% confidence level) are in bold.

| Driver | Snag | | CWD | |
| --- | --- | --- | --- | --- |
|  | Fire | Harvest | Fire | Harvest |
| LiveDBHcv | 1.0, 0.2, 0.63 | 1.4, 0.9, 0.51 | 1.8, 3.9, **0.03** | 1.581, 0.6, 0.5 |
| ISV | 1.0, 1.3, 0.26 | 1.0, 18.8, **<0.001** | 1.9, 28.0, **<0.001** | 1.0, 64.5, **<0.001** |
| LiveBA | 1.6, 4.9, **0.01** | 1.9, 12.2, **<0.001** | 1.0, 16.4, **<0.001** | 1.8, 25.5, **<0.001** |
| Site (random) | 2.0, 0.3, 0.69 | 2.0, 2.9, 0.08 | 2.0, 1.6, 0.21 | 2.0, 0.4, 0.65 |
|  |  |  |  |  |
| Deviance (%) | 40.2 | 68.7 | 84.9 | 91.0 |
